# Plasma metabolites associated with physiological and biochemical indexes indicate the effect of caging stress on mallard ducks (*Anas platyrhynchos*)

**DOI:** 10.5713/ab.21.0241

**Published:** 2021-08-25

**Authors:** Chao Zheng, Yan Wu, Zhen Hua Liang, Jin Song Pi, Shi Bin Cheng, Wen Zhuo Wei, Jing Bo Liu, Li Zhi Lu, Hao Zhang

**Affiliations:** 1 Institute of Animal Husbandry and Veterinary Science, Hubei Academy of Agricultural Sciences/Hubei Key Laboratory of Animal Embryo Engineering and Molecular Breeding, Wuhan, 430064, China; 2 School of Life Science and Engineering, Southwest University of Science and Technology, Mianyang, 621010, China; 3 Institute of Animal Husbandry and Veterinary Medicine, Zhejiang Academy of Agricultural Sciences, Hangzhou, 310021, China

**Keywords:** Caging Stress, Mallard Duck, Plasma Index, Plasma Metabolites, Production Performance

## Abstract

**Objective:**

Cage rearing has critical implications for the laying duck industry because it is convenient for feeding and management. However, caging stress is a type of chronic stress that induces maladaptation. Environmental stress responses have been extensively studied, but no detailed information is available about the comprehensive changes in plasma metabolites at different stages of caging stress in ducks. We designed this experiment to analyze the effects of caging stress on performance parameters and oxidative stress indexes in ducks.

**Methods:**

Liquid chromatography tandem mass spectrometry (LC/MS-MS) was used to determine the changes in metabolites in duck plasma at 5 (CR5), 10 (CR10), and 15 (CR15) days after cage rearing and traditional breeding (TB). The associated pathways of differentially altered metabolites were analyzed using Kyoto encyclopedia of genes and genomes (KEGG) database.

**Results:**

The results of this study indicate that caging stress decreased performance parameters, and the plasma total superoxide dismutase levels were increased in the CR10 group compared with the other groups. In addition, 1,431 metabolites were detected. Compared with the TB group, 134, 381, and 190 differentially produced metabolites were identified in the CR5, CR10, and CR15 groups, respectively. The results of principal component analysis (PCA) show that the selected components sufficiently distinguish the TB group and CR10 group. KEGG analysis results revealed that the differentially altered metabolites in duck plasma from the CR5 and TB groups were mainly associated with ovarian steroidogenesis, biosynthesis of unsaturated fatty acids, and phenylalanine metabolism.

**Conclusion:**

In this study, the production performance, blood indexes, number of metabolites and PCA were compared to determine effect of the caging stress stage on ducks. We inferred from the experimental results that caging-stressed ducks were in the sensitive phase in the first 5 days after caging, caging for approximately 10 days was an important transition phase, and then the duck continually adapted.

## INTRODUCTION

Ducks are the most widely domesticated waterfowl, and are mainly bred in China [1,2]. With the development of society and technology, the production model of laying ducks has changed from small-scale free-range farming to large-scale intensive farming. Intensive animal husbandry improves the soil utilization rate and production efficiency, reduces water pollution and prevent foreign diseases. Currently, three main methods are used to raise ducks: cage rearing, netting bed rearing and dry rearing. Each method has distinctive characteristics and results in good egg-laying performance throughout the laying period [3]. Cage rearing of ducks has great potential for development, such as automatic feeding and convenient excrement collection. However, during cage rearing, duck behavior is restricted, and behavioral restriction induces maladaptation to stressors [4]. Stress responses in poultry are undesirable, since the stress response decreases body weight, decreases food intake, and leads to economic losses [5,6]. Currently, reducing the economic losses caused by stress is still a main objective in the farming of ducks.

Chronic stress in animals is divided into two stages, the sensitive phase and the adaptable phase. As a major abiotic stress that negatively affects duck performance, caging stress is a nonpathological chronic stress, and tolerance to caging stress strongly depends on the stress response abilities of ducks. Physiological adaptations can be manifest at many levels, and activation of the hypothalamic–pituitary–adrenal (HPA) axis induces many biological mechanisms to re-establish homeostatic conditions and maintain physiological activity [7,8]. Other signaling pathways are also involved in the stress response. Stress has been hypothesized to increase the production of reactive oxygen species (ROS) in many tissues and organs, which potentially cause oxidative stress [9]. Excessive accumulation of active oxygen radicals inside the body alters gene expression in the liver and triggers metabolic changes. Moreover, ROS impair intestinal barrier function, which leads to bacterial translocation from the gut and an inflammatory reaction induced by bacterial endotoxin [10–13]. Although environmental stress responses have been extensively studied, less is known about the process of recovery, particularly recovery signals, and recovery mechanisms under different stress conditions.

Metabolites in the blood participate in the stress response and related functions by activating or deactivating related pathways. Metabolomics has been used to systematically identify changes in metabolites in organisms and elucidate their responses to environmental changes, growth or various diseases. Metabolomics has provided new perspectives to investigate the evolution of stress responses, and mass spectrometry is a good approach for discovering new biomarkers. Stress studies have identified a variety of metabolites associated with food intake, egg production, and body weight, but those metabolites that play physiological functions in the transition from the stress sensitive phase to the adaptable phase have received less attention.

As a new breeding model, few reports have examined biomarkers of cage-rearing stress in ducks. Based on this background, the physiological conditions of the caging-stress response of ducks were investigated by measuring production performance and plasma biochemical indexes after the ducks were placed in cages. For this experiment, we used untargeted metabolomics based on liquid chromatography-mass spectrometry (LC-MS) to investigate metabolite perturbations and to identify associated metabolic pathways and plasma biomarkers. The purposes of this study were to provide valuable biochemical information, to determine the mechanisms of chronic stress responses in cage-reared ducks, and to define biomarkers to assess cage-rearing stress in ducks.

## MATERIALS AND METHODS

### Ethical approval and consent to participate

The authors confirm that the ethical policies of the journal, as noted on the journal’s author guidelines page, have been adhered to and that appropriate ethical review committee approval has been received. The methods were conducted according to the Guidelines for Experimental Animals established by the Ministry of Science and Technology. All procedures involving animal subjects were approved by the Animal Ethics Committee of Hubei Academy of Agricultural Sciences. The authors confirm that they have followed EU standards for the protection of animals used for scientific purposes. We obtained written informed consent to use the animals in our study from the owner of the animals.

### Animals and experimental design

Sixty female 120-day-old mallards (*Anas platyrhynchos*, Lihu Duck Farm, Hubei Province) weighing 1,280 to 1,620 g were used. The experiment was designed to include random assignment to 2 processes, namely, cage-rearing treatment and floor rearing, and 5 repetitions (n = 6). In the trial group, the ducks were raised in a stepped duck coop, which was welded together by steel wire. The dimensions of the cages were 40 cm long, 60 cm wide and 50 cm high, and 2 ducks were placed in one cage. The ducks were raised under a light cycle of 18 hours of light and 6 hours of darkness. All ducks were fed the same diet, which met the requirements. The amount of feed provided to each bird was 160 g/d. An automatic water supply system provided clean water for the birds.

### Measurement of production performance

Food intake and the number of eggs laid were recorded daily for each group. The body weights were determined on the first day of the experiment and at 5-day intervals (days 5, 10, and 15) using an electronic balance.

### Sample preparation for MS analysis and biochemical index determination

Samples of fasting venous blood were collected from the wing veins of ducks in the following 4 stages: traditional breeding (TB), 5 days after cage rearing (CR5), 10 days after cage rearing (CR10), and 15 days after cage rearing (CR15). Then, at various time points, the selected ducks were immediately anesthetized with sodium pentobarbital (intraperitoneal injection: 150 mg/kg) and euthanized by exsanguination. The blood sample was collected in a tube containing anticoagulant and centrifuged at 3,000×g and 4°C for 10 min to obtain the plasma, which was flash frozen in liquid nitrogen, and all samples were quickly stored at −80°C until further processing.

### Determination of malondialdehyde, superoxide dismutase, and glutathione peroxidase levels

The plasma levels of superoxide dismutase (SOD), glutathione peroxidase (GSH-PX), and malondialdehyde (MDA) were determined with clinical chemistry assay kits according to the manufacturer’s instructions (Nanjing Jiancheng Bioengineering Institute, Nanjing, China). The SOD, MDA, and GSH-PX contents were determined at 550 nm, 532 nm, and 412 nm, respectively, by performing a quantitative colorimetric analysis. We calculated the concentrations of these substances based on the optical density and the standard curve according to the formula provided in the instructions.

### Sample preparation for the liquid chromatography tandem mass spectrometry analysis

The plasma samples (100 μL) were sent to Novogene (Tianjin, China) for an untargeted metabolomics analysis. The samples were individually resuspended in prechilled methanol and 0.1% formic acid by vortexing well. The samples were incubated on ice for 5 min and then centrifuged at 15,000 rpm at 4°C for 5 min. A portion of the supernatant was diluted to a final concentration of 60% methanol with LC-MS grade water. The samples were subsequently transferred to a fresh Eppendorf tube with a 0.22 μm filter and then centrifuged at 15,000×g for 10 min at 4°C. Finally, the filtrate was injected into the liquid chromatography tandem mass spectrometry (LC-MS/MS) system for analysis.

Equal volumes of each experimental sample were mixed and marked as quality control (QC) samples. The blank sample was the blank matrix of the experimental sample, and the pretreatment process for the blank sample was the same as that for the experimental samples.

LC-/MS-MS analyses were performed using a Vanquish UHPLC system (Thermo Fisher, Waltham, MA, USA) coupled to an Orbitrap Q Exactive HF-X mass spectrometer (Thermo Fisher, USA). Samples were injected onto a Hyperil Gold column (100×2.1 mm, 1.9 μm) using a 16 min linear gradient at a flow rate of 0.2 mL/min. The eluents for positive polarity mode were eluent A (0.1% formic acid in water) and eluent B (methanol). The eluents for negative polarity mode were eluent A (5 mM ammonium acetate, pH 9.0) and eluent B (methanol). The solvent gradient was set as follows: 2% B, 1.5 min; 2% to 100% B, 12.0 min; 100% B, 14.0 min; 100% to 2% B, 14.1 min; and 2% B, 16 min. A Q Exactive HF-X mass spectrometer was operated in positive/negative polarity mode with a spray voltage of 3.2 kV, a capillary temperature of 320°C, a sheath gas flow rate of 35 arb and an aux gas flow rate of 10 arb.

#### Statistical data analysis

The raw data files generated by ultra-high performance liquid chromatography–tandem mass-spectrometry (UHPLC-MS/MS) were processed using Compound Discoverer (CD) 3.0 software to perform peak alignment, peak picking, and quantitation for each metabolite. Afterwards, the peak intensities were normalized to the total spectral intensity. The normalized data were used to predict the molecular formula based on additive ions, molecular ion peaks and fragment ions. Then peaks were matched with the mzCloud (https://www.mzcloud.org/) and ChemSpider (http://www.chemspider.com/) databases to obtain the accurate qualitative and relative quantitative results. The significance of the difference between groups was analyzed according to metabolite expression levels. Principal component analysis (PCA), partial least squares-discriminate analysis (PLS-DA), variable importance in projection (VIP ≥1.0), and fold change analysis (fold change ≥2 or ≤0.5 and p-value <0.05 was considered a significant difference) were performed as described in a previous report. Then, Kyoto encyclopedia of genes and genomes (KEGG) pathway analyses (http://www.genome.jp/kegg/) of differentially expressed compounds were performed. The most enriched pathway terms with p-values <0.05 in the CR vs TB comparison were selected.

### Statistical analysis

Differences between two groups were analyzed using SPSS software with a two-tailed Student’s t-test. Differences were considered significant when p<0.05. All data are presented as the means±standard deviations.

## RESULTS

### Effect of cage-rearing on body weight gain, feed intake and the egg-laying rate

As shown in Table 1, compared with the TB groups, ducks in the CR groups showed a significant decrease in body weight and feed intake in the first 5 days, which started to increase over the next 10 days. The weights of the CR groups were significantly lower than those of the TB group. Compared with TB ducks, ducks in the CR groups exhibited a sustained decrease in the egg-laying rate. Additionally, mortality was not observed in any of the tested groups.

### Plasma levels of oxidative stress indexes

As shown in Table 2, no significant differences in GSH-PX and MDA levels were observed between the ducks in the TB group and those in the CR groups. The plasma levels of total superoxide dismutase (T-SOD) were increased in the CR10 group compared with the other groups.

### Quality control, principal component analysis, and metabolite profile analysis

**Figure 1:**
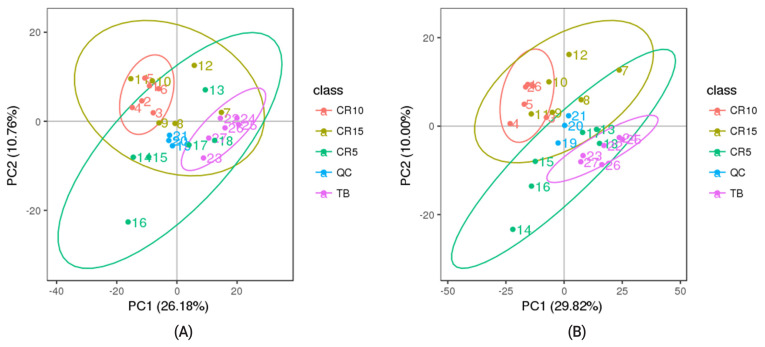
Principal component analysis of QC samples. Quality control samples (QC) can be gathered together and the better aggregation indicated that the instrument more stable and the quality of data collected. The X-axis represents the first principal component, and the Y-axis represents the second principal component. Different colour point represents different samples (n = 6) in each group. (A) positive model, (B) negative model.

**Figure 2:**
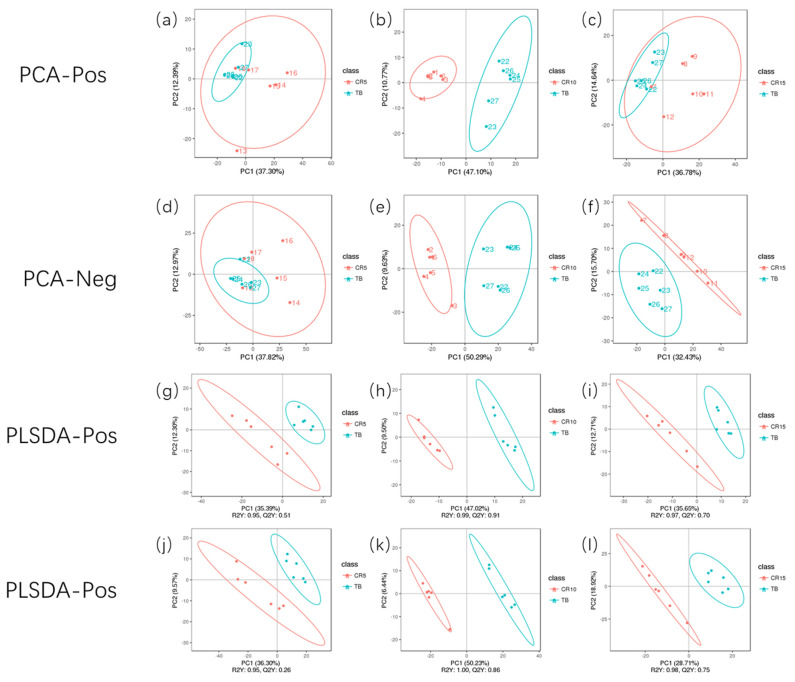
Principal component analysis model (PCA) and PLS-DA model of serum metabolites. (a–f) were PCA score plots of serum samples collected from 120-day-old ducks at caged 5 days, 10 days, 15 days and traditional breeding ducks in positive and negative models; (g–l) were PLS-DA score plots in positive and negative models. Each point in the graph represents one sample, and the discretization of the two-colour symbols represents the distribution of the two sets of samples on the PC1 and PC2 axes respectively. PLS-DA, partial least squares-discriminate analysis.

As shown in Figure 1, the QC samples were clustered in the PCA score plots, indicating that the mass spectrometry system was stable and that the quality of the data collected in this study was good. According to the PCA results, the samples in ellipsoids were distinguished from each other, thereby indicating a net diversity of metabolites at different stages of stress in CR ducks (Figure 2a-f). Based on the feature annotation described in the methods, 1,431 metabolites were identified, 662 of which were identified in positive mode and 769 of which were identified in negative mode.

### Identification of significantly altered metabolites

The number of significantly altered metabolites identified in multiple analyses is shown in Table 3. We compared metabolites with significant differences among the CR5, CR10, CR15, and TB groups to elucidate the roles of metabolites in the stress responses of ducks. Levels 134 metabolites were significantly changed in duck plasma between the TB group and the CR5 group, and levels 72 of these metabolites were significantly increased. Moreover, the concentrations of 381 metabolites were significantly different in duck plasma between the TB group and the CR10 group, and levels of 189 of these metabolites were significantly increased. The levels of 190 metabolites were significantly different in duck plasma between the TB group and the CR15 group, and the levels of 119 of these metabolites were significantly increased. These results are shown in Supplementary Tables S1–S3.

### KEGG differential metabolite enrichment analysis

**Figure 3:**
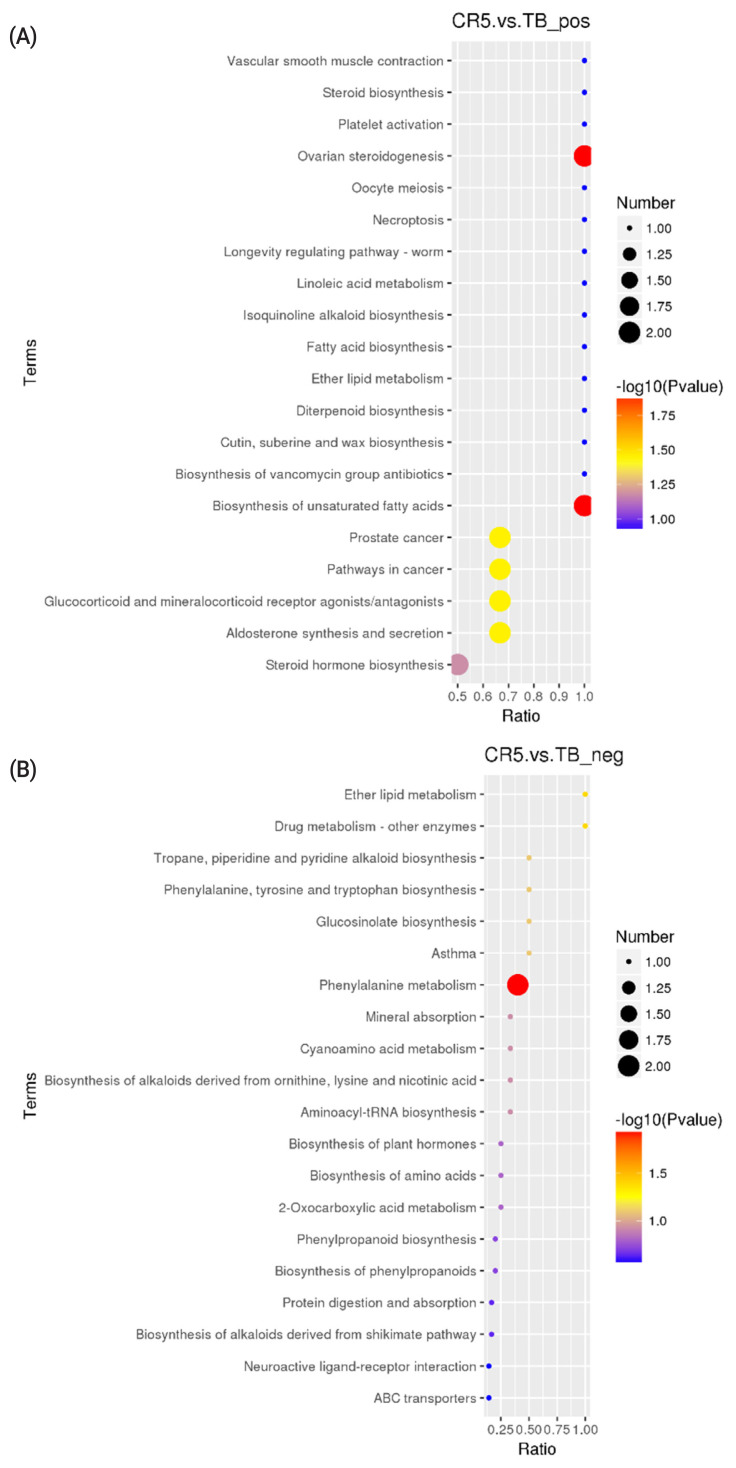
KEGG Pathway enrichment scatterplot. (A) The disturbed pathways in CR5 group vs TB group in positive model. (B) The disturbed pathways in CR5 group vs TB group in positive negative model. The circle size and color reflect the significance of the perturbed pathway. KEGG, Kyoto encyclopedia of genes and genomes; TB, traditional breeding; CR5, 5 days after cage rearing.

**Figure 4:**
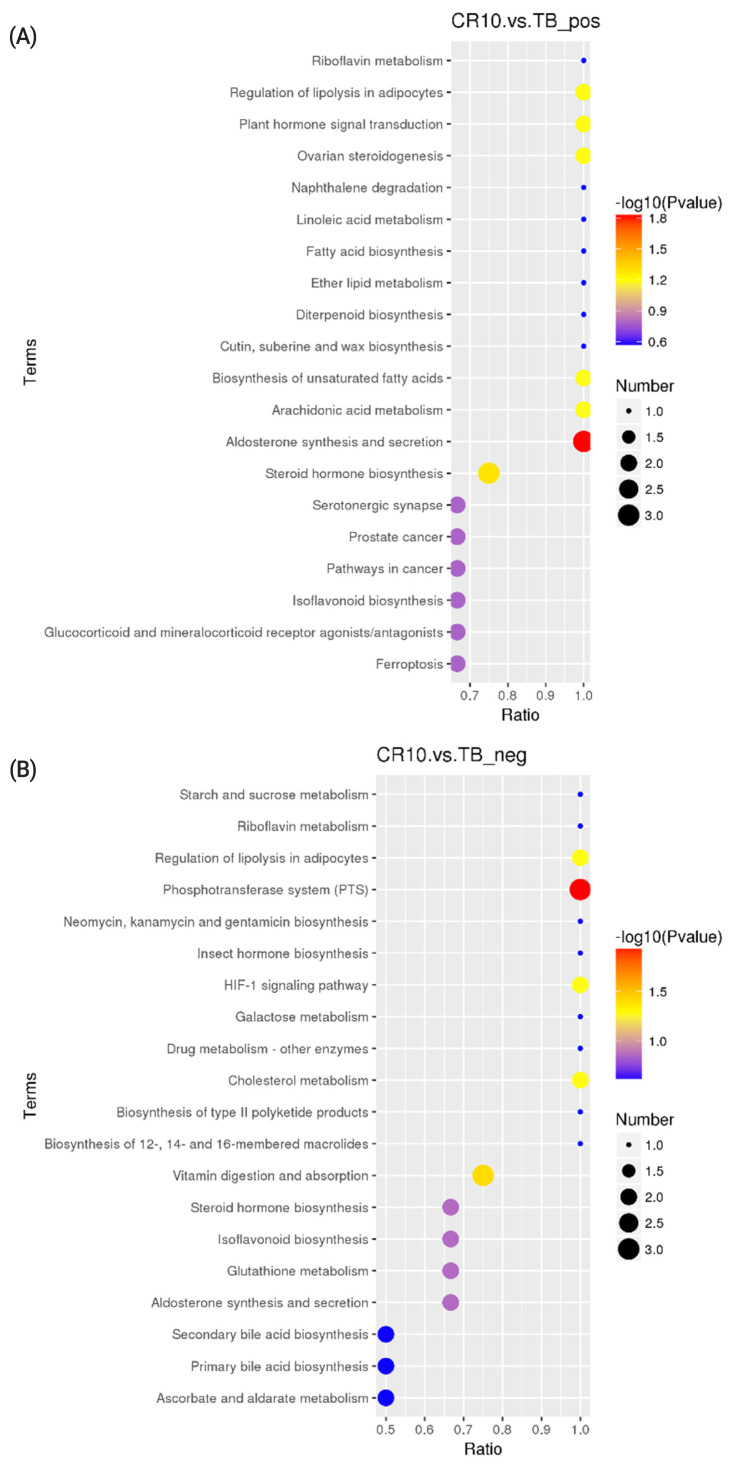
KEGG pathway enrichment scatterplot. (A) The disturbed pathways in CR10 group vs TB group in positive model. (B) The disturbed pathways in CR10 group vs TB group in positive negative model. The circle size and color reflect the significance of the perturbed pathway. KEGG, Kyoto encyclopedia of genes and genomes; TB, traditional breeding; CR10, 10 days after cage rearing.

**Figure 5:**
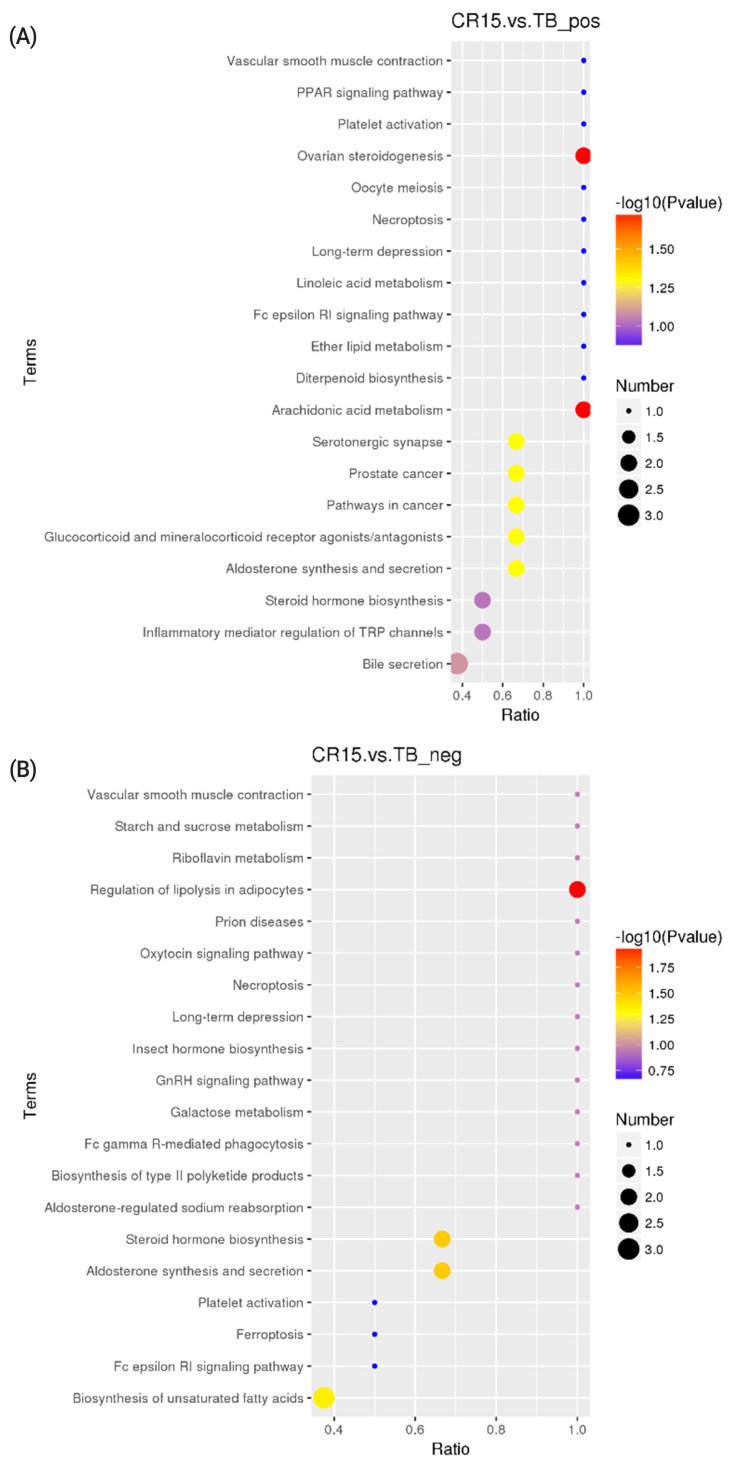
KEGG Pathway enrichment scatterplot. (A) The disturbed pathways in CR15 group vs TB group in positive model. (B) The disturbed pathways in CR15 group vs TB group in positive negative model. The circle size and color reflect the significance of the perturbed pathway. KEGG, Kyoto encyclopedia of genes and genomes; CR5, 5 days after cage rearing; TB, traditional breeding.

KEGG analysis results revealed that the differentially altered metabolites in the duck plasma of the CR5 and TB groups were mainly associated with ovarian steroidogenesis (arachidonic acid and progesterone), biosynthesis of unsaturated fatty acids (arachidonic acid), and phenylalanine metabolism (N-acetyl-L-phenylalanine and L-phenylalanine) (Figure 3a, 3b). The differentially metabolites in the duck plasma from the CR10 and TB groups were mainly associated with the phosphotransferase system (PTS) (ascorbic acid), aldosterone synthesis and secretion (arachidonic acid and corticosterone), vitamin digestion and absorption (vitamin B2 [VB2], ascorbic acid, and retinoic acid), and steroid hormone biosynthesis (progesterone, cortisol, and corticosterone) (Figure 4a, 4b). Additionally, the differential metabolites in the duck plasma from the CR15 and TB groups were mainly associated with ovarian steroidogenesis (arachidonic acid, progesterone), arachidonic acid metabolism (leukotriene B4, docosahexaenoic acid [DHA], and eicosapentaenoic acid [EPA]), regulation of lipolysis in adipocytes (arachidonic acid, corticosterone) (Figure 5a, 5b).

## DISCUSSION

Modern production management and breeding technology may lead to stress reactions. As a new breeding model, cage rearing of ducks increases productivity and serves as a tractable model for studying adaptation to stress in animals. The production performance of livestock and poultry is altered during the initial response to stress. Our research shows that caged rearing decreased the duck body weight and food intake in the first 5 days and gradually increased in the subsequent 10 days, with statistically significant differences. Lei et al [14] reported that immune stress caused by vaccination programs decreases the food intake and average daily weight gain of broilers [14]. Moreover, some studies reported that production performance is suppressed during heat stress [15,16]. However, the stress exposure period increases, animal adaptability continuously improves. Bergoug et al [17] reported that the transportation duration affects the body weight of newly hatched broilers, an effect that lasted until 21 days. Pickering et al [18] reported that after at least 7 days, flies adapt to repeated stresses. Bekenev et al [19] showed that weaned piglets adjust to inadequate environmental factors and that an additive can accelerates recovery.

The investigation of changes in blood indexes of caging-stressed ducks provides a theoretical basis for revealing the different stages of duck caging stress. No obvious changes in the levels of MDA and GSH-PX in plasma were observed after caging, the plasma T-SOD levels increased significantly after 10 days. Changes in organisms during stress are postulated to be linked to accumulating lipid peroxidation products, such as free radicals, peroxy radicals, hydroperoxides, aldehydes, and ketones [19]. Antioxidants and lipid peroxidation are key factors in the response to stress. Lipid peroxidation is defined as a parameter of oxidative stress [20]. T-SOD acts as a catalyst in the removal of toxic superoxide radicals [21]. An elevated level of T-SOD activity is a defense mechanism against an increased level of free radicals under stressful conditions [22]. The increased T-SOD activity in the CR10 group suggests that this time point potentially represents an important transition phase.

When stress occurs, animals have been shown to activate predicable, adaptive responses to stress in many systems, including the metabolic, cardiovascular, neuroendocrine, and even digestive systems [23]. Plasma metabolites include a variety of biochemical components, and changes in these metabolites reflect the responses of the animal to environmental stress [24]. We adopted an LC-MS/MS method to quantitatively study plasma metabolites and further investigate the mechanism underlying the response of poultry to chronic stress. In this study, 1,431 metabolites were detected, and 134, 381, and 190 differentially produced metabolites were identified in the CR5, CR10, and CR15 groups, respectively, compared with the TB group. To some extent, the number of differentially produced metabolites reflects the response of the body to stress. The comparison between the CR10 group and the TB group revealed the largest number of differentially produced metabolites, indicating that the organism showed a positive compensatory response to caging stress.

Based on acquired differentially abundant metabolites, PCA was performed to investigate the presence of intrinsic clusters within the data matrix. The PCA showed a striking pattern in plasma samples clustered according to the experimental timepoint, with a progressive shift from the TB group through the CR5, CR10, and CR15 groups. An obvious distinction between the TB groups and CR10 groups in positive mode and negative mode was observed, representing the inherent metabolic difference between these two groups. The PCA revealed the clustering of samples, which can be used as a method to identify different states and has been showed in many studies that applied metabolomics methods. According to the PCA results from the study by Cui et al [25] LC-MS/MS distinguishes between patients with breast cancer and healthy subjects [25]. Hendrickson et al [26] used PCA-based ellipsoid clustering to show a progressive shift from preburn samples to 72 hours postburn injury samples in a porcine model.

A KEGG pathway enrichment analysis was carried out on the acquired differentially abundant metabolites. Several pathways or biological processes were identified and may provide insights into the underlying response mechanism of caging stress. Ovarian steroidogenesis, and biosynthesis of unsaturated fatty acids were significantly different between ducks reared without cages and ducks in the CR5 group. L-Phenylalanine, N-acetyl-L-phenylalanine, platelet-activating factor (PAF), arachidonate, oleic acid, progesterone and cortisol are the key metabolic compounds in the aforementioned metabolic pathways. Significant changes in intermediates involved in the PTS, aldosterone synthesis and secretion, vitamin digestion and absorption, and steroid hormone biosynthesis were observed in ducks from the CR10 group ducks compared with the TB groups. Arachidonate, corticosterone, progesterone, ascorbate vitamin C (VC), retinoic acid, and VB2 are key metabolic compounds involved in these metabolic processes. Ovarian steroidogenesis, arachidonic acid metabolism, and regulation of lipolysis in adipocytes were significantly different between ducks reared without cages and ducks in the CR15 group. Arachidonate, leukotriene B4, progesterone, corticosterone, cortisol, DHA, and EPA are key metabolic compounds involved in the aforementioned metabolic processes. The metabolites detected in this study showed that, the differential metabolites in the caging-stress groups were strongly associated with steroid hormone biosynthesis and regulation, lipid metabolism and oxidation-reduction reaction signaling pathways. Steroid hormones participate in organ development, reproduction, body homeostasis, and stress responses [27]. Because of their anti-inflammatory properties, steroids have been proposed as therapeutic adjuvants in systemic inflammation [28]. During the caging process of laying ducks, there is a certain degree of oxidative stress reaction, and the body produces an antioxidant tissue response, and the metabolites we found were related to the anti-inflammatory function. The differentially altered metabolites identified in our experiment deserve further study.

N-acetyl-L-phenylalanine, L-phenylalanine, PAF, and oleic acid showed significant differences in metabolite ratios between the CR5 and TB groups. Aromatic amino acids, particularly L-phenylalanine, are known to increase plasma cholecystokinin levels and reduce food intake in humans [29]. The abnormal expression of L-phenylalanine and its derivatives is associated with a reduction in food intake during the early stage of stress. PAF is a potent phospholipid messenger that exerts a distinct spectrum of biological and pharmacological effects [30] and is involved in the physiological response to psychological stress [31]. The change in oxidative stress in multiple target organs of ducks may be one of the important reasons for the increase in PAF induced by caging stress. The increase in oleic acid may be related to the induction of oxidative stress.

The metabolic ratios of VC, riboflavin (VB2), and retinoic acid were significantly different between the CR10 and TB groups, and these metabolites participate in the process of oxidative stress and lipid peroxidation. The VC is an antioxidant because it prevents other compounds from being oxidized [32]. The VC acts synergistically with other vitamins in the management of oxidative stress [33]. The VB2 inhibits oxidative stress, especially lipid peroxidation and oxidative injury [34]. Ashoori et al [35] reported that VB2 protects the body from oxidative stress by inducing glutathione redox cycling, in addition to conversion of reduced VB2 to the oxidized form. The contents of endogenous VC and VB2 were significantly increased in the CR10 group compared with the TB group, which may be closely related to the increasing activity of T-SOD. Retinoic acid is a derivative of vitamin A (retinol). The endogenous tretinoin content was significantly decreased in the CR10 group compared with the TB group, which may convert the oxidized form to the reduced retinol by endogenous antioxidants. Retinoic acid can influence epithelial cell activity and might be helpful in repairing stress-mediated injured tissues [36]. Laying ducks are at the peak of oxidative stress, the vitamin consumption of the body increases, and the content of the serum decreases, which is not conducive to the maintenance of cell function and the recovery of the body.

Significant differences in the ratios of the metabolites leukotriene B4, DHA, and EPA were observed between the CR15 and TB groups, and these metabolites play major roles in the anti-inflammatory process and in regulating the viscosity of cell membranes [37]. These findings reflect the physiological changes in ducks during the caging-stress response. From stress sensitivity to antioxidant and anti-inflammatory activities that help repair and protect cells, caged ducks increasingly adapt to their new living surroundings.

## CONCLUSION

In summary, in this study, the production performance, blood indexes, number of metabolites and PCA were compared to determine the caging stress stage of ducks. We inferred from the experimental results described above that caging-stressed ducks were in the sensitive phase in the first 5 days post after caging, caging for approximately 10 days was an important transition phase, and then the duck continually adapted. Metabolic signatures were presented and biomarkers were defined to evaluate the stress status of caged ducks. This research can serve as the basis for further investigations of duck caging stress and provides a new perspective for studying the mechanism of animal stress.

## Figures and Tables

**Table 1:** Effects of caging stress on the performance parameters of ducks

Items	TB^1)^			CR^1)^		
	
	0 to 5 d	5 to 10 d	10 to 15 d	0 to 5 d	5 to 10 d	10 to 15 d
BWG (g)	37.67±66.63	14.58±78.84	34.58±79.01	−74±92.09^*^^b^	22.5±73.79^a^	24.21±88.02^a^
FI (g)	159.47±1.34	159.24±1.426	159.18±1.93	129.13±10.35^*^^b^	133.6±7.84^*^^b^	140.4±6.57^*^^a^
LR (%)	88.67±7.93	93.07±9.95	93.8±11.66	56±11.67^*^^a^	39.8±12.95^*^^b^	26.67±14.23^*^^c^

Results presented as mean±standard deviation of five replicates (n = 6).

BWG, body weight gain; FI, food intake; LR, laying rate; ANOVA, analysis of variance.

1) In cage-rearing groups (CR), 6 ducks were randomly selected every 5 days, and in traditional-breeding groups (TB), 6 ducks were randomly selected in 8 days, the selected ducks were sampled, and the other ducks’ production performance were measured continue.

Parametric data: two-way ANOVA followed by a t-test and two-way ANOVA followed by a t-test.

* p<0.05 compared with the TB groups in the same period.

a–c Different superscript letters in same line indicate significant difference among cage-rearing groups (p<0.05).

**Table 2:** Effects of caging stress on the plasma indexes about oxidative stress of ducks

Parameter	Group TB^1)^	Group CR5^1)^	Group CR10^1)^	Group CR15^1)^
T-SOD (U/mL)	84.71±44.17^a^	87.20.59±55.29^a^	152.19±15.28^b^	89.28±37.41^a^
GSH-PX (μg/mL)	329.47±230.83	374.74±157.88	412.63±102.53	371.58±151.47
MDA (nmol/mL)	8.68±2.16	7.65±2.81	8.03±3.58	7.48±1.13

Results presented as mean±standard deviation (n = 6).

SOD, superoxide dismutase; GSH-PX, glutathione peroxidase; MDA, malondialdehyde; ANOVA, analysis of variance.

1) TB, traditional breeding; CR5, 5 days after cage rearing; CR10, 10 days after cage rearing; CR15, 15 days after cage rearing.

Parametric data: two-way ANOVA followed by a T-test.

a,b Values with different letters are different (p<0.05).

**Table 3:** The analysis results of significant metabolites which identified by LC-MS/MS

Compared samples^1)^	Num. of total Ident.^2)^	Num. of total Sig.^3)^	Num. of Sig.Up^4)^	Num. of Sig.down^5)^
CR5.vs TB_pos	662	75	54	21
CR10.vs TB_pos	662	187	114	73
CR15.vs TB_pos	662	92	62	30
CR5.vs TB_neg	769	59	18	41
CR10.vs TB_neg	769	194	75	119
CR15.vs TB_neg	769	98	57	41

LC-MS/MS, liquid chromatography tandem mass spectrometry.

1) TB, traditional breeding; CR5, 5 days after cage rearing; CR10, 10 days after cage rearing; CR15, 15 days after cage rearing.

2) Num. of total Ident., number of total identified metabolites.

3) Num. of total Sig., number of total significant metabolites.

4) Num. of Sig.Up, number of total significant metabolites which be up-regulated.

5) Num. of Sig. down, number of total significant metabolites which be down-regulated.

Base on variable important for the projection (VIP ≥1.0), fold change ≥2 or ≤0.5, and p-value <0.05.

## SUPPLEMENTARY MATERIAL

**Supplementary Table S1.** Differentially plasma metabolites at CR5 when compared to that those at TB in ducks **Supplementary Table S2.** Differentially serum metabolites at CR10 when compared to that those at TB in ducks **Supplementary Table S3.** Differentially serum metabolites at CR15 when compared to that those at TB in ducks
