# Supplementary material for: Plasma metabolites associated with physiological and biochemical indexes indicate the effect of caging stress on mallard ducks (Anas platyrhynchos)
Source: Anim Biosci. 2021 Aug 25;35(2):224–35. doi: 10.5713/ab.21.0241 (PMC8738941; doi:10.5713/ab.21.0241)
Supplement: Supplementary file 1 [file ab-21-0241-suppl.pdf]

Table S1. Differentially plasma metabolites at CR5 when compared to that those at TB in ducks.

Descrption	Mode	RT[a]	CR5	TB	FC[b]	P-value	AUC[c]	VIP[d]
1-arachidonoyl-sn-glycerol 3-phosphate	neg	14.29	363992.81±110752	171005.07±59584	2.129	1.75E-03	0.97	1.04
1-Palmitoyl lysophosphatidic acid	neg	14.48	502319.42±170894	183954.21±67352	2.731	1.44E-03	0.94	1.92
20-Oxopregn-5-en-3-yl hydrogen sulfate	neg	12.39	8251.97±7016	55064.27±56898	0.15	4.35E-03	0.97	2.57
2-arachidonoylglycerol	neg	12.39	9295.21±3620	76671.35±40215	0.121	2.97E-05	1	2.98
4-(2-Aminophenyl)-2,4-dioxobutanoic acid	pos	7.53	22532.41±9268	77865.34±29147	0.289	1.28E-03	0.97	2.05
5-Methoxydimethyltryptamine	neg	13.85	69737.44±64566	234487.01±66481	0.297	8.20E-03	0.97	1.97
7-ketodeoxycholic acid	neg	12.39	1097138.82±536015	12262345.58±7461117	0.089	2.08E-05	1	3.46
Arachidonic acid	pos	15.07	525267.87±329553	145584.7±27963	3.608	7.47E-03	0.97	1.69
Calcitriol	pos	14.04	44938.24±24079	17851.11±5440	2.517	9.79E-03	0.97	1.34
Clomipramine	pos	15.34	65720.81±28861	30137.59±9400	2.181	8.77E-03	0.92	1.11
Cortisone	pos	15.35	442753.53±201897	138591.22±55583	3.195	1.24E-03	1	1.72
Cystathionine	pos	1.21	140949.1±86765	43654.03±11548	3.229	7.99E-03	0.94	1.59
D (+)-Phenyllactic acid	neg	5.32	179132.7±61390	483437.08±173339	0.371	9.24E-04	1	1.35
Ethopabate	neg	7.89	41583.73±20402	125690.1±64744	0.269	7.05E-03	0.94	1.78
Ethopabate	pos	9.56	41583.73±20402	125690.1±64744	0.269	7.16E-03	0.92	1.61
Furomine	pos	15.44	13962.27±5544	6085.85±2277	2.294	3.47E-03	0.94	1.25
Gibberellin A4	pos	15.13	57634.17±28130	16295.66±9655	3.537	4.15E-03	0.94	1.94
Indaconitine	pos	15.21	111456.95±53718	33790.84±7185	3.298	5.35E-03	1	1.66
IpA	neg	7.97	99512.08±71447	550660.57±519783	0.181	8.34E-03	0.97	2.19
L-Phenylalanine	neg	3.8	10684745.21±4319171	23379697.38±8558084	0.457	5.28E-03	0.97	1.02
Lucel	neg	11.13	1974.22±401	11544.25±7895	0.171	2.31E-03	1	2.34
Methyl indole-3-acetate	pos	10.34	132328.26±70329	588822.37±458884	0.225	3.43E-03	1	2.18
Militarinone A	pos	12.75	37808.79±33762	135230.75±59856	0.28	2.78E-03	0.94	2.22

Myristyl sulfate	pos	15.34	4322.25±2119	6362.13±5001	2.546	3.67E-03	0.94	1.37
Oleoyl-L-α-lysophosphatidic acid	neg	14.6	161800.03±57098	61705.63±30541	2.622	1.50E-03	0.94	1.74
Oxagrelate	neg	7.77	4962.2±2908	17208.14±9225	0.288	3.90E-03	0.94	1.62
PALGLY	neg	14.44	111853.94±57115	33086.29±18187	3.381	9.37E-03	0.92	1.47
Sunitinib	neg	12.82	14119.68±13239	53236.59±19528	0.265	7.02E-03	0.94	2.18
Terpendole J	pos	15.19	45738.53±17916	9890.95±5107	4.624	1.85E-04	0.97	2.46

[a] Retention time (min). [b] Fold change at CR5 as compared with TB. [c] Area Under Curve. [d] Variable important for the projection. We just listing out the metabolites that the p-value < 0.01 here.

Table S2. Differentially serum metabolites at CR10 when compared to that those at TB in ducks.

Descrption	Mode	RT[a]	CR10	TB	FC[b]	P-value	AUC[c]	VIP[d]
(-)-citrinin	pos	6.05	23335.27±11240	98540.71±26472	0.24	7.68E-05	1	2
(-)-Epothilone D	neg	13.2	14884.55±9482	145441.26±77542	0.1	8.45E-05	1	2.3
(+)-Lacosamide	pos	7.16	13404.11±5732	32149.73±11807	0.42	4.46E-03	0.92	1.18
(+)-Nootkatone	neg	11.15	24824.18±11825	79714.77±37985	0.31	1.76E-03	0.97	1.15
(10S)-Juvenile hormone III diol	pos	13.47	72121.6±38672	18961.41±5328	3.8	2.68E-03	0.97	1.64
(2E)-hexadecenoylcarnitine	pos	13.05	382767.38±109852	100834.75±86833	3.8	3.55E-03	1	2.15
(2R)-2,3-Dihydroxypropyl 18-methylicosanoate	neg	14.08	52683.07±33157	8966.45±3630	5.88	4.14E-04	1	1.68
(3E)-4,8-Dimethyl-1,3,7-nonatriene	neg	12.71	194673.39±111602	62925.68±37019	3.09	7.68E-03	0.94	1.22
(E)-3,4,5-Trimethoxycinnamic acid	neg	8.84	14024.41±6417	56357.93±13396	0.25	1.87E-04	1	1.47
(E,Z)-Gloxazone	pos	1.35	17864.96±3938	42464.14±12574	0.42	1.29E-03	0.94	1.13
(Z)-Endoxifen	pos	15.34	65641.71±16679	24261.82±8564	2.71	2.77E-04	1	1.38
10-HDA	neg	11.26	163723.26±60276	666532.6±165193	0.25	1.17E-05	1	1.42
12(S)-HHT	neg	12.78	78424.43±58915	17472.25±6446	4.49	7.55E-03	0.97	1.31
12,22-Dihydro-2H,3H-porphine	pos	14.97	59998.04±26497	18560.97±10317	3.23	4.24E-03	0.92	1.6
14(Z)-Eicosenoic acid	neg	15.38	20961682.82±9083042	7567354.98±4635226	2.77	5.36E-03	0.92	1.15
16-Hydroxyhexadecanoic acid	neg	13.24	1646365.21±489115	433611.11±190894	3.8	2.93E-04	1	1.38
16-ketoestrone	neg	9.6	2787.2±2643	12435.04±6423	0.22	4.82E-03	0.94	1.54
1-arachidonoyl-sn-glycero-3-phosphocholine	pos	14.51	39615013.76±13295836	8996025.52±3332084	4.4	4.27E-05	1	2
1-arachidonoyl-sn-glycerol 3-phosphate	neg	14.29	897167.99±176729	171005.07±59584	5.25	3.37E-06	1	1.68
1-Palmitoyl lysophosphatidic acid	neg	14.48	843496.1±297961	183954.21±67352	4.59	4.40E-05	1	1.63
1-stearyl estercitric acid	neg	13.27	295187.64±230406	46401.43±26738	6.36	4.67E-04	1	1.87
20-Oxopregn-5-en-3-yl hydrogen sulfate	neg	12.39	4890.23±6215	55064.27±56898	0.09	3.52E-04	0.97	2.49
2-arachidonoylglycerol	neg	12.39	8880.02±5112	76671.35±40215	0.12	2.93E-05	1	2.17

2-Decylfuran	neg	12.77	13599.48±8272	2740.52±317	4.96	9.42E-03	0.86	1.38
2-Hydroxy-22-methyltetracosanoic acid	neg	15.4	108521.14±100637	13784.46±5942	7.87	2.54E-03	1	1.82
2-linoleoyl-sn-glycero-3-phosphoethanolamine	pos	14.54	19824.74±3906	94498.21±74050	0.33	1.48E-05	1	1.51
2-Methyl acetoacetate	pos	1.35	39166.74±12744	83794.43±14384	0.47	7.50E-04	1	1.07
3, 5-Tetradecadiencarnitine	pos	12.25	194849.22±85636	65178.42±47724	2.99	5.59E-03	0.94	1.69
3,5-Dihydroxybenzoic acid	neg	7.11	419310.51±161885	1523144.33±522311	0.28	1.69E-04	0.97	1.29
3-Hydroxy-cis-5-tetradecenoylcarnitine	pos	12.2	53257.87±23195	14110.9±8271	3.77	4.82E-04	0.97	1.83
3-Hydroxydecanoic acid	neg	11.51	577206.55±271153	181429.03±46211	3.18	1.08E-03	1	1.09
3-Hydroxynonanoic acid	neg	15.44	152844.81±95979	13138.41±5329	11.63	4.21E-05	1	2.36
3-Hydroxytetradecanedioic acid	neg	10.35	13930.71±3167	76639.47±48329	0.18	6.54E-04	1	1.57
3-O-(alpha-L-olivosyl)oleandolide	neg	12.3	9610442.98±5591115	82747659.69±39689444	0.12	4.12E-05	1	2.18
3β-Androstanediol	neg	14.48	349688.26±162668	120960.97±19863	2.89	3.21E-03	1	1.01
4,8a-dimethyl-decahydronaphthalen-4a-ol	neg	13.26	857637.52±641976	131154.69±74111	6.54	2.62E-04	1	1.83
4-Hydroxycyclophosphamide	pos	1.24	131888.68±28381	50580.27±13086	2.61	6.69E-05	1	1.3
4-Phenolsulfonic acid	neg	5.49	1161620.14±243418	3503766.72±1275041	0.33	1.22E-04	1	1.07
4-vinylphenol sulfate	neg	8.88	51683.47±31164	166076.73±65728	0.31	5.39E-03	0.94	1.26
5,6-Dihydroxy-2-naphthalenesulfonic acid	neg	9.65	11217.14±3156	31719.47±11632	0.35	3.48E-04	1	1.02
5-Methoxy-3-indoleaceate	neg	6.28	20625.78±7971	72746.54±48951	0.28	6.40E-03	0.97	1.17
5-O-methyl embelin	neg	11.63	16176.66±14124	4667.09±1422	3.47	9.97E-03	0.97	1.05
7-ketodeoxycholic acid	neg	12.39	496631.41±156344	12262345.58±7461117	0.04	3.72E-06	1	3.12
8-oxoguanine	pos	8.31	8821.45±871	40240.49±12776	0.22	5.96E-05	1	2
9,12-Hexadecadienoylcarnitine	pos	12.77	70682.26±27564	24375.56±17781	2.9	3.14E-03	0.92	1.6
9-Phenyl-1-(2,4,6-trihydroxyphenyl)-1-nonanone	pos	15.06	436100.09±84371	170591.06±46678	2.56	9.08E-05	1	1.28
Acetylcarnitine	neg	7.42	29964.68±11602	229103.48±128031	0.13	2.62E-04	1	1.93
Acetylcarnitine	pos	9.43	29964.68±11602	229103.48±128031	0.24	5.79E-04	0.97	1.82
Acetyl-L-methionine	pos	6.88	10978.59±3219	29011.23±12444	0.38	4.41E-03	0.94	1.23

Adenosine monophosphate	pos	1.36	37291.34±21279	13033.93±4291	2.86	8.82E-03	0.92	1.29
Aklavinone	neg	8.15	8648.73±6709	46061.95±15399	0.19	1.77E-04	1	1.79
Aliskiren	pos	15.67	209520.76±32001	85488.12±32244	2.45	1.17E-03	1	1.31
all-cis-4,7,10,13,16-Docosapentaenoic acid	pos	15.28	20502365.44±11459084	12443514.94±3280445	2.95	9.95E-03	0.97	1.29
Amiloxate	neg	11.11	13714.6±2718	100120.75±50158	0.14	2.45E-04	1	1.87
Apigetrin	neg	7.94	3062.41±506	9230.33±4910	0.33	3.13E-03	1	1.03
Arachidonic acid	neg	14.54	83932950.52±15540224	30170372.85±8119273	2.78	1.41E-05	1	1.04
Arachidonyl trifluoromethyl ketone	pos	14.99	5293.04±1312	25898.23±26101	0.2	9.22E-03	0.97	1.75
Ascorbic acid	neg	1.3	2919091.97±1469810	539776.78±650478	5.41	7.10E-03	0.94	2.38
Atropine	neg	12.32	10416.72±4986	74680.71±66307	0.14	1.12E-03	1	1.81
Avenein	neg	9.09	5077.53±5130	44383.11±45899	0.11	8.00E-04	0.97	2.12
Azidocillin	neg	6.13	1516.01±380	11911.78±6452	0.13	2.71E-05	1	1.98
belfosdil	neg	12.47	8346.54±7825	34578.57±19115	0.24	5.15E-03	0.94	1.6
Benzoxazolone	neg	9.05	125545.79±78122	593688.81±219920	0.21	1.01E-03	1	1.67
Biotin l-Sulfoxide	pos	5.84	8013.4±3554	25806.49±6264	0.31	8.93E-05	1	1.61
Butopyronoxyl	neg	9.74	72036.43±81526	4533.88±827	15.89	3.03E-03	1	2.31
Cabaser	neg	12.28	6440.4±1533	22225.58±9882	0.29	5.94E-04	1	1.18
Cannabigerolic acid	neg	12.93	450179.79±344079	104946.9±80888	4.29	5.12E-03	0.92	1.41
Cefozopran	neg	12.33	35781.53±30034	295279.92±123103	0.12	2.00E-04	1	2.28
Clomipramine	neg	12.99	3387.93±2267	10420.35±4447	0.33	6.37E-03	0.94	1.17
Clonixeril	neg	1.39	69765.91±37940	365115.84±118312	0.19	2.17E-05	1	1.69
Clonixin	neg	9.49	227504.67±216966	2943.41±405	77.29	3.75E-03	1	3.66
Corticosterone	pos	11.68	47286.86±37979	150591.11±69324	0.29	5.62E-03	1	1.9
Cortisol	neg	10.87	6682.36±4982	27099.68±16099	0.25	2.06E-03	0.97	1.51
Cortisone	pos	15.35	596774.76±236248	138591.22±55583	4.31	1.06E-03	0.94	1.9
Cuauhtemone	neg	12.77	64802.41±44291	4922.66±1189	13.16	7.50E-03	0.92	2.17

Cucurbitacin p	neg	13.68	18851.55±10954	2756.29±376	6.84	3.87E-04	1	1.83
cystathionine	pos	1.21	174826.66±80704	43654.03±11548	4	1.83E-04	1	1.8
Daidzein	neg	8.82	30090.28±14481	368288.04±190937	0.08	2.26E-05	1	2.5
Daidzein	pos	9.51	30090.28±14481	368288.04±190937	0.15	1.87E-05	1	2.51
Daidzein-7-O-glucuronide	pos	8.28	4845.2±2606	30487.76±24355	0.16	3.87E-03	0.97	2.21
Decursin	neg	14.5	15202.34±8774	3266.93±1149	4.65	1.78E-03	1	1.47
Desoxymycin	neg	12.19	20872.52±13949	77846.2±35923	0.27	4.09E-03	0.97	1.53
Dianthramine	neg	12.51	3053.54±573	21496.48±16282	0.14	8.90E-03	1	1.75
Dihydrojasmone	neg	10.96	211908.36±124377	2743.67±979	77.24	4.44E-03	0.92	3.76
Dihydrostreptomycin	neg	12.02	8358.12±7228	31427.59±17216	0.27	1.53E-03	0.92	1.42
Di-n-Amyl phthalate	neg	11.75	69979.6±23179	21325.91±14829	3.28	8.80E-04	0.97	1.27
Disperse orange 3	neg	8.3	27779.07±31718	104234.3±16713	0.27	3.07E-03	0.94	1.65
Djenkolic Acid	pos	1.24	391602.44±117334	136081.45±31755	2.88	1.51E-04	1	1.4
Docosahexaenoic acid	neg	14.49	18456266.54±6892288	5356901.91±1557434	3.45	2.27E-04	1	1.22
Docosatetraenoic acid	pos	15.45	151890.8±102936	29569.79±16376	5.14	6.71E-04	1	2.14
Docosatrienoic acid	neg	15.23	1485982.07±704641	527633.63±316626	2.82	9.23E-03	0.94	1.19
D-pantothenic acid	pos	5.99	1351286.01±190455	3169703.53±499234	0.43	3.69E-06	1	1.14
Eicosapentaenoic acid	neg	14.28	1511191.27±567472	397103.64±120301	3.81	8.77E-05	1	1.32
Epelsiban	neg	12.07	3648.48±897	20801.27±14998	0.18	7.34E-04	1	1.59
Epothilone C	neg	12.82	60445.78±23810	659479.58±705580	0.09	5.30E-04	1	2.12
Eprazinone	neg	12.32	5642.77±1364	23429.41±9638	0.24	1.15E-04	1	1.37
Ethopabate	pos	9.56	25204.56±12392	291196.38±162452	0.17	3.11E-04	1	2.27
Ethyl eicosapentaenoic acid	pos	15.17	51448.93±16529	30810.76±22111	3.18	3.34E-03	1	1.45
Ethyl vanillin propylene glycol acetal	pos	11.57	12799.3±2444	20817.22±13634	0.21	9.79E-04	1	2.02
Fasoracetam	pos	6.52	41722.35±15007	196257.12±98822	0.31	1.27E-03	1	1.47
Formiminoglutamic Acid	pos	1.33	22075.92±4722	54980.43±29454	0.4	2.76E-03	1	1.15

Gamma-glutamyltyramine	neg	8.19	2557.78±1413	25648.39±11489	0.1	2.12E-05	1	2.28
Genistein	pos	10.11	53312.93±23814	375958.3±212532	0.39	8.06E-04	1	1.2
Geranylacetone	neg	13.24	20476.59±8293	5357.96±2782	3.82	4.82E-04	0.97	1.39
Germine	neg	13.03	13833.17±7083	97406.61±50797	0.14	8.23E-05	1	1.94
Gibberellin A4	pos	15.13	87161.56±26630	16295.66±9655	5.35	2.72E-04	1	2.39
Glycocholic acid	neg	12.42	7166.13±1281	77075.22±74167	0.09	4.32E-03	1	1.97
Gly-l-pro	pos	1.36	51094.18±6798	129202.51±60323	0.4	4.84E-03	1	1.15
Hexadecan-1-ol	neg	14.66	24745.5±12481	7687.04±2310	3.22	1.31E-03	1	1.11
Hexitol	neg	1.36	115490.36±22403	385172.31±124939	0.3	1.15E-04	1	1.17
Hexyl 2-furoate	pos	11.39	26667.01±11896	35600.78±11332	20.09	4.94E-03	0.94	3.41
Homosalate	pos	12.29	41617.85±16874	52099.69±11815	0.39	3.22E-04	1	1.24
Hydroprene	neg	14.35	870556.02±313586	234787.07±154463	3.71	2.19E-03	1	1.48
Hydroxychloroquine	pos	15.46	121472.48±29473	57109.5±27079	2.13	8.54E-03	1	1.16
Icomucret	neg	14.08	66935.06±31156	23329.83±7653	2.87	5.34E-04	1	1.02
Idoxifene	pos	15.22	18079.37±6589	7522.2±3045	2.4	5.07E-03	0.94	1.25
Indaconitine	pos	15.21	127147.32±20665	33790.84±7185	3.76	6.11E-07	1	1.81
Integerressine	neg	13.1	3730.77±1230	19321.79±9228	0.19	4.44E-03	0.94	1.51
IpA	neg	7.97	65849.81±26798	550660.57±519783	0.12	1.55E-03	1	1.87
Juniperic acid	pos	13.89	138690.51±72400	50946.94±14272	2.72	3.28E-03	1	1.26
Juvenile hormone III	neg	13.22	76944.39±25359	23769.79±7584	3.24	6.49E-04	0.97	1.15
L-alpha-Glycerylphosphorylcholine	pos	15.22	59014.86±8143	20397.99±1783	2.89	2.03E-07	1	1.42
L-Cystine	pos	1.22	2984424.54±547978	1395553.37±149116	2.14	3.63E-05	1	1.02
L-dihydroanticapsin	pos	5.99	62435.99±16463	154933.3±26644	0.4	3.81E-05	1	1.23
Leucoline	pos	10.74	29191.44±3483	83654.95±45086	0.35	2.87E-03	1	1.29
Leukotriene B4	pos	14.02	55375.49±19863	37626.84±24719	2.54	8.26E-03	0.92	1.17
Linoleyl carnitine	pos	13.2	806900.26±404379	258771.02±171431	3.12	7.66E-03	0.97	1.77

Loxoprofen	neg	10.77	21171.74±11541	136998.72±91157	0.15	8.06E-04	1	1.81
Lucel	neg	11.13	1383.83±298	11544.25±7895	0.12	8.39E-04	1	1.93
Lumichrome	pos	9.98	236504.45±300658	870701.77±230095	0.27	4.78E-03	0.89	2.3
Makisterone A	neg	13.69	16218.54±10909	2891.96±479	5.61	1.15E-03	1	1.61
Marbofloxacin	neg	14.07	2627.86±452	16387.64±7562	0.16	5.53E-04	1	1.72
Meclizine	neg	14.16	3460.59±1685	1234.5±139	2.8	2.56E-03	1	1.03
Melanostatin	neg	15.33	30598.92±3703	145192.26±88182	0.21	4.51E-03	1	1.4
Mephenesin	neg	8.73	1507373.72±952057	14031.81±3986	107.43	4.15E-03	0.92	4.02
Methionine sulfoxide	pos	1.27	444005.69±211396	1166560.17±336232	0.38	1.49E-03	0.97	1.36
Methyl indole-3-acetate	pos	10.34	115569.49±43193	588822.37±458884	0.2	1.45E-03	1	2
Methylarsonic acid	pos	1.03	86637.76±29916	22812.58±10136	3.8	1.21E-04	1	1.85
Midodrine	neg	6.35	3919.05±943	22316.58±10775	0.18	2.28E-04	1	1.65
Militarinone A	pos	12.75	19073.97±4545	135230.75±59856	0.14	8.23E-05	1	2.52
Monocrotophos	pos	8.88	2776.28±743	15661.16±9087	0.18	3.85E-03	0.97	2.08
Monodesethylchloroquine	neg	10.73	8989.5±6406	26601.25±11071	0.34	4.63E-03	0.97	1.22
Mupirocin	neg	12.91	4419374.15±1847210	38781602.11±24046781	0.11	7.02E-05	1	2.09
Mycalamide A	neg	12.02	26476.73±23276	132585.3±59883	0.2	8.63E-04	1	1.79
Myristyl sulfate	pos	15.34	43775.21±97759	6362.13±5001	2.62	4.74E-04	1	1.35
N-Acetyl-DL-tryptophan	neg	7.45	66055.05±44413	232709.85±114394	0.28	1.15E-03	0.94	1.35
N-Acetyl-L-phenylalanine	neg	7.26	31488.09±7061	101543.27±38260	0.31	1.07E-04	1	1.14
Naratriptan	pos	15.45	65535.72±21868	20474.99±11352	3.2	1.29E-03	1	1.69
Navenone A	pos	12.09	71450.49±8266	159870.13±43231	0.45	7.27E-04	1	1.06
N-feruloylglycine	pos	8.09	7500.07±4390	24597.81±9460	0.3	1.91E-03	1	1.71
Nikethamide	pos	9.23	5727.87±1507	18315.8±4756	0.31	1.39E-05	1	1.57
Norethindrone	pos	13.78	37133.27±18300	12229.09±4871	3.04	3.70E-03	0.94	1.41
Octadecane	neg	14.42	596557.82±222762	139202.28±103043	4.29	5.19E-03	1	1.73

O-glutaroyl-L-carnitine	pos	1.43	84982±24767	33524.99±11959	2.53	8.29E-04	1	1.31
O-heptanoylcarnitine	pos	9.22	20666.92±13149	5012.12±1633	4.12	8.76E-03	0.86	1.69
Oleic acid	pos	15.46	974294102.76±219790718	486561837.55±293924813	2.32	8.63E-03	0.92	1.1
Omarigliptin	neg	8.13	4050.51±3145	15277.31±4492	0.27	6.62E-04	0.97	1.46
O-oleoylcarnitine	pos	13.43	2506438.56±815665	787403.9±481446	3.18	2.77E-03	1	1.73
Oxagrelate	neg	7.77	2834.76±1905	17208.14±9225	0.16	2.43E-04	0.97	1.79
Oxybuprocaine	neg	12.21	2728.44±1432	65109.21±20914	0.04	1.27E-07	1	3.24
Palgly	neg	14.44	135399.91±84118	33086.29±18187	4.09	4.01E-04	1	1.39
Palmitoylcarnitine	pos	13.34	1118039.14±393993	407751.2±177294	2.74	1.36E-03	1	1.42
pentamidine	pos	15.35	33371.03±10269	10709.03±6621	3.12	3.71E-03	0.94	1.73
Perfluorooctanoic Acid	neg	7.52	3597.43±1738	26049.17±16415	0.14	8.32E-05	1	1.97
Petromyzonol sulfate	neg	11.86	14851.35±8669	61681.48±49379	0.24	7.51E-03	0.94	1.36
Platelet-activating factor	pos	15.21	167710.08±52057	70852.37±27207	3.33	1.16E-05	1	1.66
Plinabulin	pos	15.13	1380978.74±527418	414821.53±268564	3.33	3.32E-03	0.97	1.8
propamidine	pos	15.34	291155.85±102795	93134.94±38108	3.13	5.15E-04	1	1.58
Prostaglandin A1 ethyl ester	neg	12.47	25455.06±15978	261178.02±93392	0.1	1.34E-05	1	2.41
Protoverine	neg	12.39	3720.51±2150	43904.15±23632	0.08	5.77E-06	1	2.48
Pyrogallol	pos	2.24	1472278.98±403161	387274.08±138935	3.8	3.15E-05	1	1.81
Quinoline	pos	10.34	120638.94±47321	649206.01±492523	0.19	9.43E-04	1	2.1
Riboflavin	neg	8.15	306771.35±366773	1479711.54±499353	0.21	1.29E-03	0.97	1.9
Ricinoleic Acid	pos	14.16	13590.14±3459	9284.33±2537	2.59	9.29E-03	1	1.18
Rolofylline	pos	15.46	57178.57±20375	21982.82±8952	2.6	1.69E-03	1	1.33
Samandarin	pos	13.18	9289.9±670	22817.97±5079	0.41	8.17E-05	1	1.19
Schisandrin C	pos	15.01	21752.1±10280	5334.85±866	4.08	1.49E-03	1	1.74
Seratrodast	pos	15.46	651676.5±240019	93504.95±54179	6.97	1.28E-04	1	2.75
Sinapinic acid	pos	8.87	12066.66±4990	45552.99±29285	0.26	3.87E-03	0.97	1.64

Sucrose	neg	1.38	71996.59±15230	274363.86±77729	0.26	3.78E-06	1	1.32
Sulfoglycolithocholic acid	neg	12.66	236985.91±169138	3188137.31±893093	0.07	2.67E-05	1	2.73
Sulfolithocholic acid	neg	12.99	3417.39±1393	14627.82±11922	0.23	3.31E-03	0.97	1.29
Sulfosalicylic Acid	pos	2.24	72123±21433	22194.66±13631	3.25	1.72E-03	0.97	1.71
Sunitinib	neg	12.82	8378.11±14525	53236.59±19528	0.16	1.69E-03	0.94	2.56
Taurochenodeoxycholic acid	neg	12.91	23465439.24±9563393	169325332.44±80716214	0.14	5.95E-05	1	1.93
terameprocol	neg	12.59	67631.79±46127	10248.05±9487	6.6	6.87E-04	0.97	1.98
Terpendole J	pos	15.19	33905.15±15382	9890.95±5107	3.43	5.22E-04	1	1.73
Thienodolin	neg	8.87	16704.82±12257	73188.28±62734	0.23	4.96E-03	1	1.51
Thioinosinic acid	neg	8.9	20974.96±8044	112314.79±40014	0.19	4.55E-05	1	1.7
Trans-2-Tetradecenoylcarnitine	pos	12.6	1107916.78±430311	276218.9±251253	4.01	3.75E-03	0.97	2.24
Trigonelline	pos	1.32	200116.61±53924	496245.03±139795	0.4	2.23E-04	1	1.21
Val-Ser	pos	1.36	17796.52±3259	40959.55±8722	0.43	3.82E-05	1	1.11
Vargulin	pos	15.34	63100.81±20114	22000.98±8803	2.87	5.23E-04	1	1.46
α-Eleostearic acid	neg	14.3	69737662.49±21358871	25124214.76±13200387	2.78	2.75E-03	0.94	1.11
α-Linolenic acid	neg	14.29	69744887.2±21357926	25126627.69±13200236	2.78	2.75E-03	0.94	1.11
Δ2-trans-Hexadecenoic acid	neg	14.41	175097051.63±42998838	53126814.91±37613440	3.3	4.89E-03	1	1.42

[a] Retention time (min). [b] Fold change at CR5 as compared with TB. [c] Area Under Curve. [d] Variable important for the projection. We just listing out the metabolites that the p-value < 0.01 here.

Table S3. Differentially serum metabolites at CR15 when compared to that those at TB in ducks.

Descrption	Mode	RT[a]	CR15	TB	FC[b]	P-value	AUC[c]	VIP[d]
(+/-)-Camphoric acid	pos	10.63	63619.8±79802	8966.45±3630	2.74	4.49E-03	0.94	1.47
(2R)-2,3-Dihydroxypropyl 18-methylicosanoate	neg	14.08	39475.01±11346	80009.66±23628	7.1	8.66E-03	0.97	2.35
(9Z)-9-Octadecenamide	pos	12.98	58135.59±22627	24261.82±8564	0.49	1.81E-03	0.97	1.22
(Z)-Endoxifen	pos	15.34	75777.91±64352	17901.43±5667	2.4	4.54E-03	0.89	1.35
1,3-Dihydroxy-2-propanyl (13Z,16Z)-13,16-docosadienoate	neg	14.14	1171747.45±521953	433611.11±190894	4.23	8.66E-03	0.94	1.88
16-Hydroxyhexadecanoic acid	neg	13.24	620942.41±387525	171005.07±59584	2.7	6.82E-03	0.92	1.42
1-arachidonoyl-sn-glycerol 3-phosphate	neg	14.29	1093954.56±508030	183954.21±67352	3.63	8.82E-03	0.94	1.68
1-Palmitoyl lysophosphatidic acid	neg	14.48	407674.07±316136	46401.43±26738	5.95	2.60E-04	1	2.56
2,3-Bis[(11Z)-11-icosenoyloxy] propyl docosanoate	pos	11.95	14970.49±3590	16954.48±10861	3.26	4.19E-03	0.94	1.99
2,6-di-tert-butyl-4-ethylphenol	neg	14.5	56603.92±19544	13784.46±5942	0.24	5.97E-03	0.94	1.88
2-Hydroxy-22-methyltetracosanoic acid	neg	15.4	1039545.24±539185	356066.94±179777	4.11	4.53E-04	0.97	2.1
2-methylbutyrylcarnitine	pos	6.89	80060.29±37353	13138.41±5329	2.92	4.63E-03	0.94	1.66
3-Hydroxynonanoic acid	neg	15.44	31298.67±20902	77865.34±29147	6.09	6.68E-05	1	2.65
4-(2-Aminophenyl)-2,4-dioxobutanoic acid	pos	7.53	254478.51±42567	120312.16±32516	0.4	8.55E-03	0.89	1.56
5-aminoimidazole ribotide	pos	1.27	8691.17±1406	40240.49±12776	2.12	2.89E-04	1	1.23
8-oxoguanine	pos	8.31	424429.91±275542	145584.7±27963	0.22	1.97E-05	1	2.49
Arachidonic acid	neg	14.54	147577.64±84179	365115.84±118312	2.25	2.40E-03	0.97	1.13
Clonixeril	neg	1.39	2104795.23±1371523	1112458.48±695000	0.4	8.98E-03	0.94	1.46
Coenzyme Q2	neg	11.76	15445.09±7589	5632.72±602	2.76	7.03E-03	0.92	1.44
Compound II(R/S)	neg	10.78	832898.86±384934	138591.22±55583	2.74	7.34E-03	1	1.31
Cortisone	pos	15.35	29797.23±21246	2756.29±376	6.01	3.20E-04	1	2.71
Cucurbitacin P	neg	13.68	140139.35±54151	43654.03±11548	10.81	1.36E-03	1	3.25
Cystathionine	pos	1.21	9247.78±5633	3266.93±1149	3.21	8.72E-04	1	1.76

Decursin	neg	14.5	49064.09±22067	18863.85±6997	2.83	9.10E-03	0.94	1.35
Dilauroyl peroxide	neg	15.37	149370.19±116020	12538.57±5837	2.6	3.26E-03	0.94	1.39
Di-n-Amyl phthalate	pos	12.79	13890246.18±4257977	5356901.91±1557434	11.91	1.14E-03	1	3.51
Docosahexaenoic acid	neg	14.49	14608.47±8428	4855.71±1752	2.59	8.28E-04	0.97	1.35
Eicosapentaenoic acid	neg	14.09	15542.92±6470	6085.85±2277	3.01	8.57E-03	0.94	1.51
Furomine	pos	15.44	72165.07±51941	16295.66±9655	2.55	2.10E-03	0.97	1.46
Gibberellin A4	pos	15.13	464246.69±378383	50742.78±29158	4.43	4.54E-03	0.94	2.23
GLYCERYL 1,2-DICAPRATE	neg	13.28	2536236.51±1044188	1089746.41±439515	9.15	2.30E-03	0.97	3.04
Hexadecanedioic acid	neg	12.31	98080.7±45845	33790.84±7185	2.33	5.20E-03	0.92	1.2
Indaconitine	pos	15.21	38293.69±17125	19996.94±6192	2.9	5.08E-03	0.94	1.58
Juvenile hormone III	neg	13.22	1111403.2±587038	606938.45±289796	2.29	4.76E-04	0.97	1.21
Kinoprene	neg	13.9	52324.1±22171	20397.99±1783	0.41	7.95E-04	1	1.33
Leukotriene B4	pos	14.023	31377.35±25973.93	8523.14±3271.76	3.68	3.32E-02	0.81	1.71
L-alpha-Glycerylphosphorylcholine	pos	15.22	55056.08±49363	7684.87±2867	2.57	5.52E-03	1	1.39
L-Hexanoylcarnitine	pos	8.09	2391.61±1276	11544.25±7895	7.16	6.77E-03	0.97	2.63
Lucel	neg	11.13	28446.49±25393	2891.96±479	0.21	2.95E-03	0.94	2.1
Makisterone A	neg	13.69	3049.32±419	16387.64±7562	9.84	4.79E-03	1	2.94
Marbofloxacin	neg	14.07	21232.18±3544	145192.26±88182	0.19	1.02E-03	1	2.27
Melanostatin	neg	15.33	17880.37±3315	135230.75±59856	0.15	1.34E-03	1	2.56
Militarinone A	pos	12.75	11478056.46±7181089	38781602.11±24046781	0.13	1.11E-04	1	3.14
Mupirocin	neg	12.91	4692.5±1179	18315.8±4756	0.3	9.22E-03	0.94	1.84
Nikethamide	pos	9.23	72075.71±23086	33524.99±11959	0.26	2.47E-06	1	2.24
O-glutaroyl-L-carnitine	pos	1.43	22657.37±13870	5012.12±1633	2.15	3.85E-03	0.94	1.24
O-heptanoylcarnitine	pos	9.22	211488.59±80370	61705.63±30541	4.52	5.25E-03	0.97	2.13
Oleoyl-L-α-lysophosphatidic acid	neg	14.6	5106.91±1737	28609.28±33215	3.43	6.28E-04	0.94	1.85
Olivomycin	pos	5.97	11102.86±2165	4458.36±940	0.18	9.27E-03	0.97	2.22

Ouabain	pos	14.76	61360.74±35823	14890.98±10855	2.49	1.70E-05	1	1.54
Paradol	pos	12.8	63996.75±7470	26616.81±4700	4.12	4.89E-03	0.94	2.3
Piromidic acid	pos	14.31	222573.55±89590	93134.94±38108	2.4	2.07E-06	1	1.43
propamidine	pos	15.34	7893.44±739	22817.97±5079	2.39	9.80E-03	0.89	1.36
Samandarin	pos	13.18	16358.36±8757	5334.85±866	0.35	1.85E-05	1	1.72
Schisandrin C	pos	15.01	630247.81±426605	93504.95±54179	3.07	9.84E-03	0.94	1.58
Seratrodast	pos	15.46	47353.36±28407	10248.05±9487	6.74	5.81E-03	0.89	2.78
Terameprocol	neg	12.59	557727.37±304791	193330.42±54611	4.62	2.56E-03	0.92	2.36
Tetradecanedioic acid	neg	11.22	28031.29±8939	9872.24±3851	2.88	5.29E-03	0.97	1.41
Traumatic Acid	pos	7.03	55895.43±19354	22000.98±8803	2.9	4.72E-03	0.94	1.65

[a] Retention time (min). [b] Fold change at CR5 as compared with TB. [c] Area Under Curve. [d] Variable important for the projection. We just listing out the metabolites that the p-value < 0.01 here.
